# Interplay between copy number alterations and immune profiles in the early breast cancer Scandinavian Breast Group 2004-1 randomized phase II trial: results from a feasibility study

**DOI:** 10.1038/s41523-021-00352-3

**Published:** 2021-11-19

**Authors:** Ioannis Zerdes, Michele Simonetti, Alexios Matikas, Luuk Harbers, Balazs Acs, Ceren Boyaci, Ning Zhang, Dimitrios Salgkamis, Susanne Agartz, Pablo Moreno-Ruiz, Yalai Bai, David L. Rimm, Johan Hartman, Artur Mezheyeuski, Jonas Bergh, Nicola Crosetto, Theodoros Foukakis

**Affiliations:** 1 Department of Oncology-Pathology, Karolinska Institutet, Stockholm, Sweden; 2 Breast Center, Theme Cancer, Karolinska University Hospital, Stockholm, Sweden; 3 Division of Genome Biology, Department of Medical Biochemistry and Biophysics, Karolinska Institutet, Stockholm, Sweden; 4 Science for Life Laboratory, Stockholm, Sweden; 5 Department of Pathology and Cytology, Karolinska University Hospital, Stockholm, Sweden; 6 Department of Pathology, Yale School of Medicine, New Haven, CT USA; 7 Department of Immunology, Genetics, and Pathology, Uppsala University, Uppsala, Sweden

**Keywords:** Breast cancer, Cancer genomics, Cancer microenvironment

## Abstract

Emerging data indicate that genomic alterations can shape immune cell composition in early breast cancer. However, there is a need for complementary imaging and sequencing methods for the quantitative assessment of combined somatic copy number alteration (SCNA) and immune profiling in pathological samples. Here, we tested the feasibility of three approaches—CUTseq, for high-throughput low-input SCNA profiling, multiplexed fluorescent immunohistochemistry (mfIHC) and digital-image analysis (DIA) for quantitative immuno-profiling- in archival formalin-fixed paraffin-embedded (FFPE) tissue samples from patients enrolled in the randomized SBG-2004-1 phase II trial. CUTseq was able to reproducibly identify amplification and deletion events with a resolution of 100 kb using only 6 ng of DNA extracted from FFPE tissue and pooling together 77 samples into the same sequencing library. In the same samples, mfIHC revealed that CD4 + T-cells and CD68 + macrophages were the most abundant immune cells and they mostly expressed PD-L1 and PD-1. Combined analysis showed that the SCNA burden was inversely associated with lymphocytic infiltration. Our results set the basis for further applications of CUTseq, mfIHC and DIA to larger cohorts of early breast cancer patients.

## Introduction

The substantial proportion of patients with breast cancer (BC) that do not respond to immunotherapy by checkpoint inhibition underscores the importance of identifying reliable predictive biomarkers. To date, the only prospectively validated marker remains Programmed Death Ligand 1 (PD-L1) protein expression, which predicts benefit to immunotherapy in metastatic triple-negative BC, albeit with contradictory results depending on the chemotherapy backbone^1,2^. Moreover, PD-L1 protein detection has been characterized by controversial analytical performance and ambiguous prognostic role in BC^3^. Other factors that describe or determine tumor-host interactions such as tumor-infiltrating lymphocytes (TILs), gene expression immune signatures and tumor mutational burden (TMB) represent markers of response to chemotherapy^4–7^, with emerging data also supporting prediction of benefit from immunotherapy^8,9^. Furthermore, genome instability, aneuploidy, as well as immune evasion have been recognized as important biomarkers of BC progression^10,11^. The interplay between tumor aneuploidy/somatic copy number alterations (SCNAs) and the immune response has been demonstrated in advanced BC and other tumor types and can impact both prognosis and therapy response^12–15^. However, little is known about the interplay between SCNAs and the patterns of immune cell infiltration in early BC.

Recent methodological advances can greatly facilitate the study of SCNAs and the immune microenvironment by using low-input clinical tumor samples. We recently developed a method, CUTseq, which enables highly multiplexed SCNA profiling at high resolution (10 kilobases, kb) even when using picogram quantities of genomic DNA (gDNA) extracted from small (4–5 mm^2^) areas in single sections of formalin-fixed paraffin-embedded (FFPE) samples^16^. In parallel to sequencing technologies for profiling aneuploidy/SCNAs, emerging automated digital imaging analysis (DIA) technologies, such as multiplexed fluorescent immunohistochemistry (mfIHC) and automated TIL enumeration now allow robust identification and quantitation of multiple immune markers even in FFPE tumor tissue sections^17^.

Here, we sought to demonstrate the applicability of CUTseq, mfIHC and DIA to FFPE BC tissue sections from patients enrolled in the Scandinavian Breast Group (SBG) 2004-1 phase II early BC trial and to portray immuno-genomic correlates detected in these samples.

## Results

### Expression patterns of immune cell subsets and related markers

**Fig. 1:**
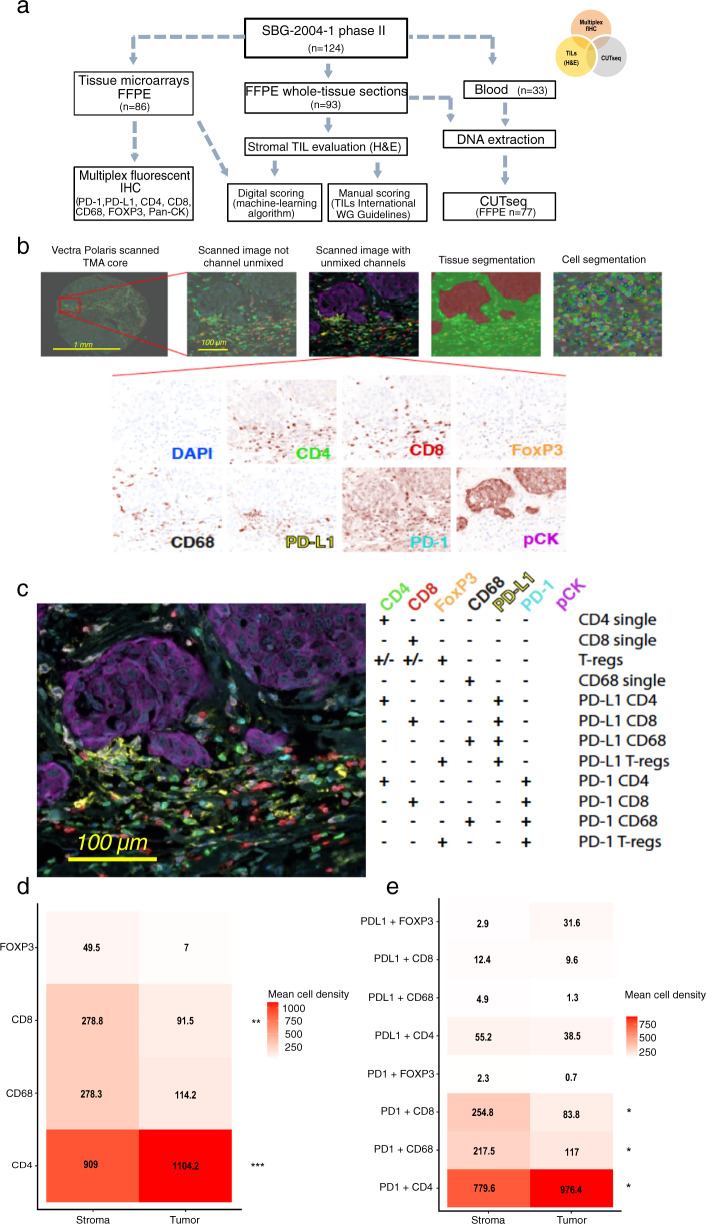
Immune profiling using a multiplexed method in early breast cancer formalin-fixed paraffin-embedded (FFPE) tissue. **a** Flowchart of the patient sample availability and methods used in the translational sub-study of the SBG-2004-1 early breast cancer trial; **b** Overview and workflow of the multiplex fluorescent IHC approach in tissue microarrays; **c** Representative image of the spatial immune cell distribution and phenotyping according to the (co)expression of the relevant markers; **d**, **e** Heatmaps depicting mean cell densities of immune cell subpopulations and expression patterns per tissue compartment (*n* = 79).

We performed mfIHC in 86 out of 124 FFPE samples (69.3%) available from patients initially enrolled in the SBG-2004-1 trial, which were previously arrayed on tissue microarrays (TMA) (Fig. 1a, b and Methods). We assessed the number and spatial location of single-positive (+) CD4 and CD8 T-cells, CD68 + macrophages and FoxP3+ regulatory T-cells as well as their combined expression with the immune checkpoint markers PD-L1 and PD-1 (Fig. 1c). Both in tumor and stromal tissue compartments of matched samples, CD4 + T-cells were the most abundant cell type (mean cell density: 1104 and 909 cells/mm^2^, respectively), followed by CD68 + macrophages (mean cell density: 114.2 and 278.3 cells/mm^2^, respectively) (Fig. 1d and Supplementary Table 1), with higher mean cell densities observed in ER-negative compared to ER-positive tumors (Supplementary Fig. 1a). PD-L1 and PD-1 were mostly expressed in CD4 + T-cells in both intra-tumoral (38.5 cells/mm^2^ and 976.4 cells/mm^2^, respectively) and stromal (55.25 cells/mm^2^ and 779.6 cells/mm^2^, respectively) areas. Stromal PD-1 + CD8 + and PD-1 + CD68 + (254.8 and 217.5 cells/mm^2^, respectively) cell subsets were also expressed at high frequency (Fig. 1e and Supplementary Table 1). Immune cell populations expressing checkpoint proteins were more abundant in ER-negative tumors (Supplementary Fig. 1b). The densities of the different intra-tumoral or stromal immune subtypes were moderately to strongly correlated (Supplementary Table 2). Nevertheless, hierarchical clustering revealed no difference between stromal or tumor-related immune cell clusters (Supplementary Fig. 2). In short, immune checkpoints molecules were expressed both in lymphocytes and macrophages, with a strong propensity for the stromal compartment.

### Manual and digital automated evaluation of tumor-infiltrating lymphocytes

**Fig. 2:**
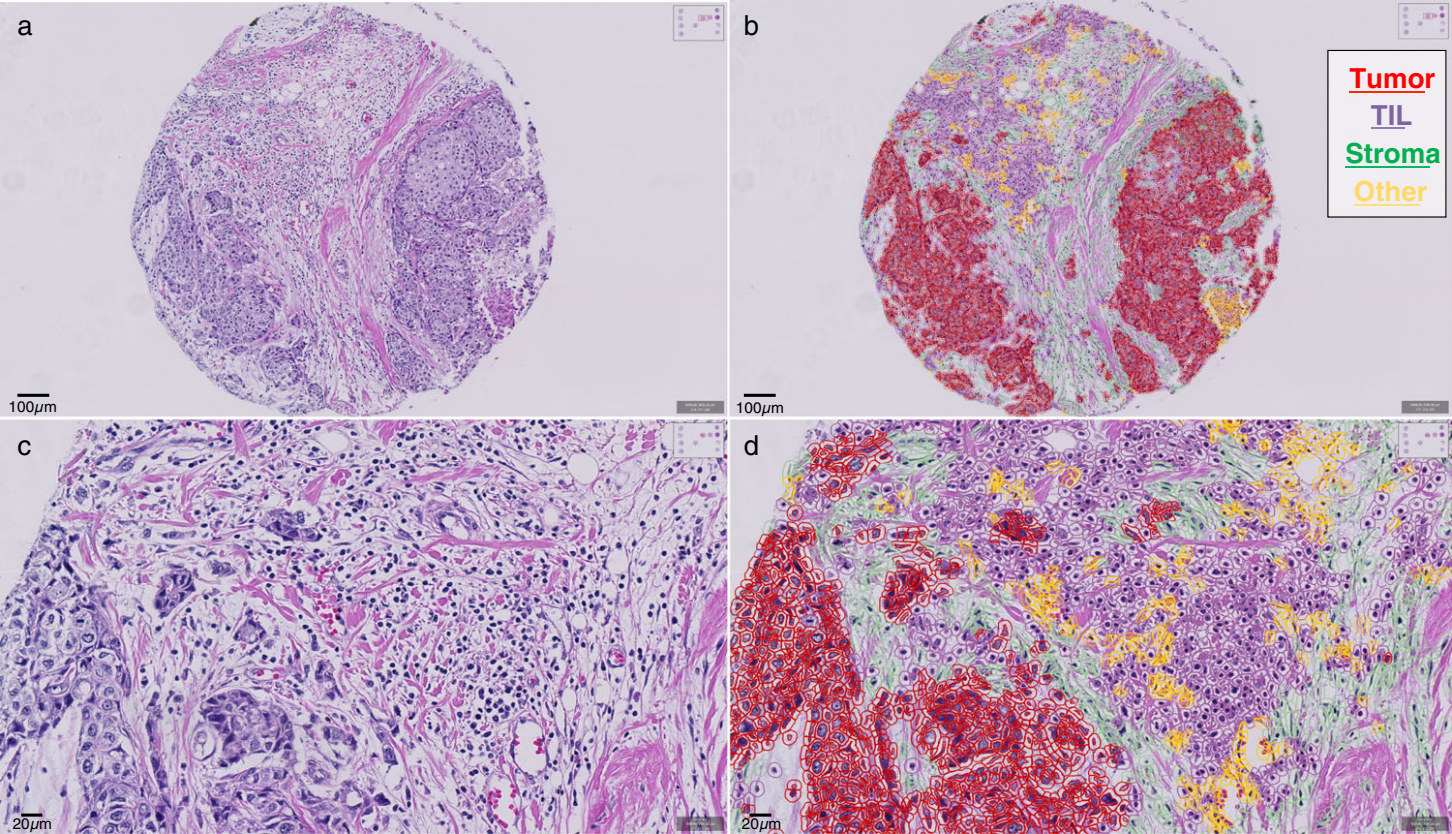
TILs manual and digital evaluation. Representative image of a tissue-microarray (TMA) core (**a** and **c,** original magnification x200 and x400, respectively); Digital image analysis and different cell type annotations in the same images, using a machine-learning algorithm (**b** and **d,** original magnification x200 and x400, respectively).

**Fig. 3:**
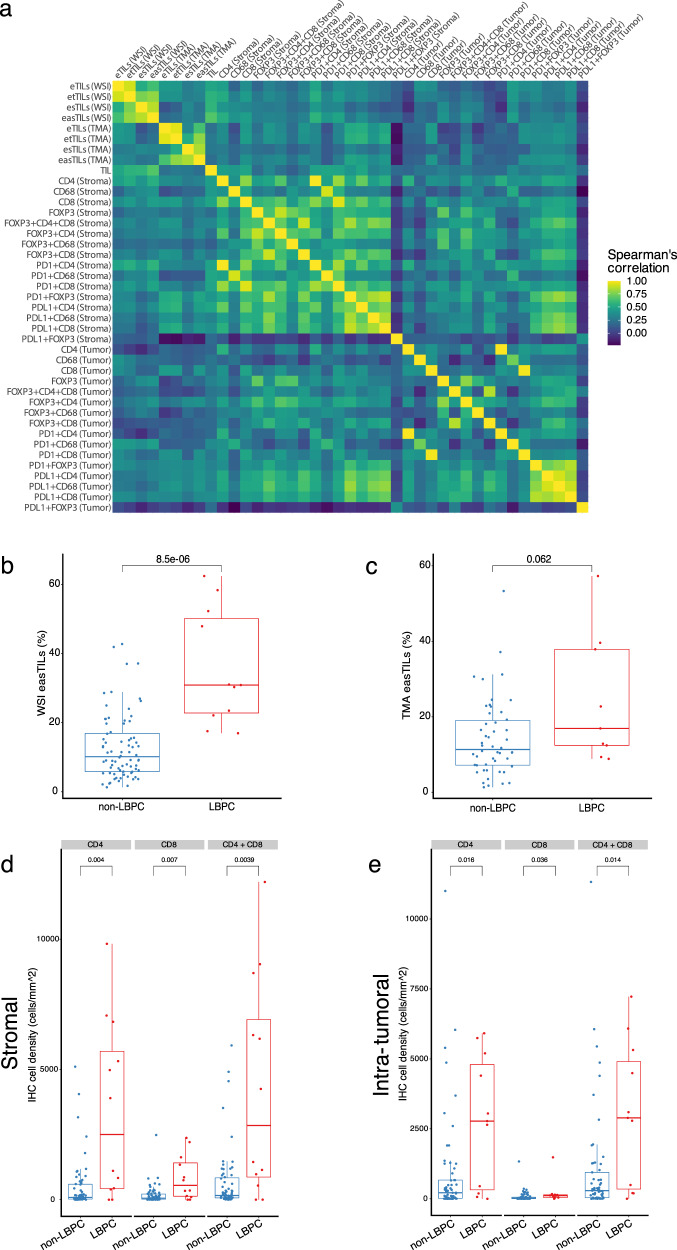
Correlations of TILs with multiplex IHC. **a** Correlation matrix of the different variables derived from multiplex fluorescent IHC, manual and digital TILs scoring; Correlations of easTILs in whole-slide images (**b**) and TMA (**c**) with manual TILs scoring in lymphocyte-predominant breast cancer (LPBC) and non-LPBC; Correlation between CD4 and CD8 stromal (**d**) and intra-tumoral (**e**) immune cell subsets with LPBC and non-LPBC based on manual TILs scoring.; In the boxplots, each box extends from the 25th to the 75th percentile, the midline represents the median, and the whiskers extend from –1.5 × IQR to +1.5 × IQR from the closest quartile, where IQR is the inter-quartile range; WSI: whole-slide images, TMA: tissue microarrays.

We then performed TIL enumeration in whole-tissue sections (WTS) stained with hematoxylin-eosin (H&E) in a total of 93 evaluable samples by eye according to International TILs/Immuno-Oncology Biomarker Working Group Guidelines. The median expression of stromal TILs was 10% (range: 1–90), and high stromal TIL levels were associated with estrogen receptor negativity (*p* < 0.001, Pearson’s chi-squared test) and high tumor grade (*p* < 0.001, Pearson’s chi-squared test) (Supplementary Table 3). We also tested the performance of an automated TIL scoring algorithm (easTILs, see Methods) in the same tissue slides both in WTS (*n* = 88) and in TMAs (*n* = 66) (Fig. 2). The median score of the easTILs digital evaluation was 12.27% (range: 1.30–62.44% of stroma area) in WTS and 12.95% (range: 1.32–65.29% of stroma area) in TMA. Manual TIL scoring was strongly and statistically significantly correlated with easTILs in WTS (Spearman’s rho = 0.677, *p* < 0.001, two-tailed) but not in TMA (Spearman’s rho = 0.210, *p* = 0.13, two-tailed) (Fig. 3a–c and Supplementary Table 2). Furthermore, automated TIL evaluation scores were not statistically significantly correlated between WTS and TMAs in patients with matched tissue (Spearman’s rho=0.263, *p* = 0.06, two-tailed) (Fig. 3a and Supplementary Table 2). In the same samples assessed by mfIHC, we also examined the correlation between CD4 + and CD8 + stromal cell densities with the extent of TIL infiltration. The mean stromal and intra-tumoral CD4 + and CD8 + T-cell density and their sum (CD4 + and CD8 +) were significantly higher in lymphocytic predominant BC samples (LPBC; stromal TIL > 50%) compared to non-LPBC tumors (Fig. 3d, e and Supplementary Table 2). Similarly, the percentages of automatically counted TILs in WTS and in TMAs were moderately correlated with CD4 + and CD8 + cell densities (Fig. 3a and Supplementary Table 2). These results show that prediction of TIL enumeration by digital counting depends on tissue source and that mfIHC-derived CD4 and CD8 densities are representative of TIL abundance.

### SCNA profiling by CUTseq

Out of 96 FFPE samples evaluated by mfIHC and/or TILs scoring, 93 samples had gDNA of sufficient quality to be used for CUTseq. As reference for calling SCNAs, we used gDNA extracted from peripheral mononucleated blood cells available for 33 patients (Methods). To assess the reproducibility of CUTseq, we sequenced two libraries prepared using two aliquots of each of the 93 gDNA samples. Forty-four samples reached a sufficient sequencing read depth in both replicates and showed a high correlation of the per bin log2 ratio up to 100 kilobase resolution (Supplementary Fig. 3a). After copy number calling, we observed a significant correlation (Pearson’s rho = 0.94; *p* = 2.2e-16, two-tailed) of the percentage of the human genome that was amplified or deleted in corresponding replicates and a high concordance of the SNCA profiles between them (Supplementary Fig. 3b, c).

**Fig. 4:**
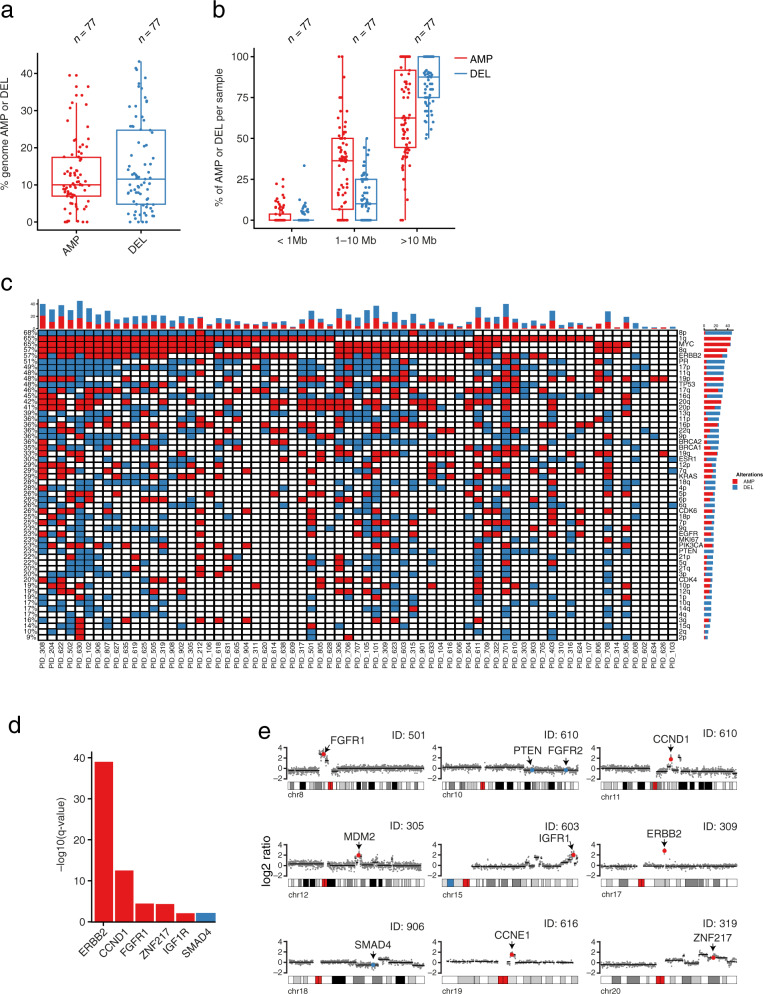
Copy number alteration distribution and frequency in FFPE samples using the CUTseq method. Percentage (**a**) and size (**b**) of the altered genome (amplified or deleted) in the SBG-2004-1 study. In the boxplots, each box extends from the 25th to the 75th percentile, the midline represents the median, and the whiskers extend from –1.5× IQR to +1.5× IQR from the closest quartile, where IQR is the inter-quartile range; **c** Landscape of the most frequently altered chromosomal arm and gene loci among study samples; **d**, **e** Significantly focally altered genes in relation to their chromosomal location.

To increase the number of samples, we merged corresponding replicates, which led to 77 out of 93 samples (82.7%) with enough sequencing reads to allow reliable SCNAs calling at 100 kb resolution (see Methods). In these samples, deletions (DEL) were present in higher percentage as compared to amplifications (AMP) (14.6% and 12.2%, respectively) (Fig. 4a). When further subdividing the samples into ER-negative and ER-positive samples we saw similar AMP and DEL percentages (12.4% and 14.9% in ER-positive samples and 11.8% and 14.1% in ER-negative samples, respectively) (Supplementary Fig. 4a). The majority of the alterations were large-sized (>10 megabases, Mb), both in the case of AMP and DEL, followed by medium-sized (1–10 Mb) and focal (<1 Mb) events (Fig. 4b). This was also the case when further stratifying samples by ER status (Supplementary Fig. 4b). AMP occurred most frequently on chromosome 1q, 8q, 17q, 20q, while DEL were predominant on 8p, 11q and 17p (Fig. 4c). We then checked which genes listed in the Catalog of Somatic Mutations in Cancer (COSMIC)^18^ were most frequently amplified or deleted. Among these, *MYC* was the most frequently altered gene locus (65% of all the samples) followed by *ERBB2* (57%), *TP53* (48%), *BRCA2* (36%) and *BRCA1* (36%) (Fig. 4c). We also used the GISTIC (Genomic Identification of Significant Targets in Cancer)^19^ algorithm to detect significantly focally altered genes. *ERBB2* was the most significantly focally altered gene, followed by *CCND1*, *FGFR1* and *ZNF217* (Fig. 4d, e). Altogether, these results show that high-throughput CUTseq allows robust detection of SCNAs in low-input clinical samples.

### Immune-genomic analyses reveal an inverse link between SCNA burden and immune response

**Fig. 5:**
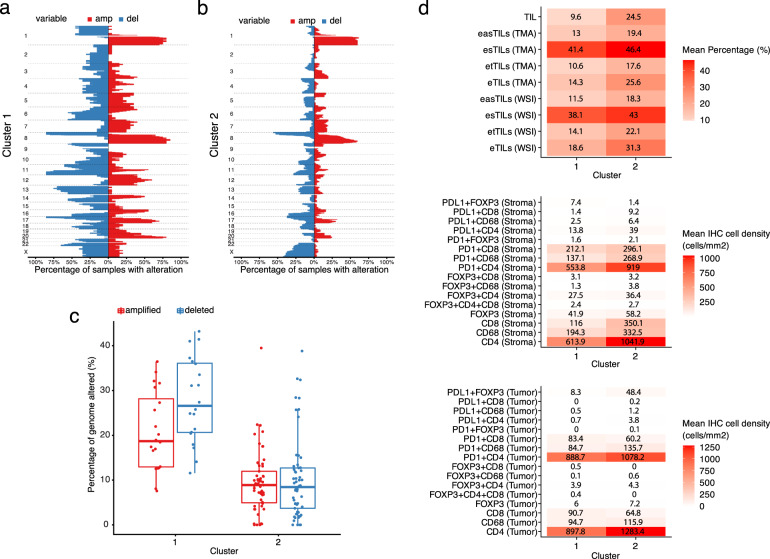
Combined immunogenomic analysis in the SBG-2004-1 trial. **a**, **b** CNA clusters and graph depicting the respective CNA burden (percentage of genome amplified/deleted); **c** Distribution of CNA among the distinct genomic clusters. In the boxplots, each box extends from the 25th to the 75th percentile, the midline represents the median, and the whiskers extend from –1.5× IQR to +1.5× IQR from the closest quartile, where IQR is the inter-quartile range; **d** Heatmaps of the mean cell density and expression of the different multiplex immune cell subsets and H&E TIL variables (both manual and digital TILs) within CNA clusters.

We then aimed at deciphering the relationship between the tumoral genomic architecture and the composition of the immune infiltrate. Using unsupervised hierarchical clustering analysis, we detected two distinct genomic groups: cluster 1 (*n* = 20) was characterized by higher SCNA burden (i.e., the percentage of the genome either amplified or deleted) as compared to cluster 2 (*n* = 57), which had a lower SCNA burden (Fig. 5a, b). We did not find any significant difference between the two clusters in terms of overall survival (two-sided log-rank *p* = 0.372). However, we did observe a differential composition of the immune cell infiltrate between the two clusters. The mean distribution of visually scored stromal TILs was significantly lower in cluster 1 as compared to cluster 2 (mean TILs percentage: 9.6% *vs*. 24.5% in cluster 1 and 2, respectively, p = 0.016, Mann–Whitney U test, two-tailed) (Fig. 5d, upper panel). Similarly, the mean percentage expression of all digital TILs variables, in both WTS and TMAs, were higher in cluster 2 (Fig. 5d, upper panel). When we evaluated specific immune cell subsets, we found that intra-tumoral CD4 + and CD68 + cells as well as stromal CD4 + , CD8 + and CD68 + cell abundances were signifiscantly increased in cluster 2 (Fig. 5d, middle and lower panels). Altogether, our results indicate that, in early breast cancer tumors, an inflamed tumor microenvironment tends to be associated with a lower SCNA burden and vice versa.

## Discussion

This is a correlative and proof-of-principle study that demonstrates the applicability of newly developed methods (CUTseq for SCNA genomic profiling and mfIHC and digital image analysis for immune profiling) in archival FFPE tissue, using samples from patients enrolled in the phase II SBG-2004-1 early BC trial. mfIHC facilitated the qualitative and quantitative assessment of several markers in a tissue-compartment manner (tumor *versus* stroma area), while digital TILs scoring^20^ enabled the evaluation of morphology-based immune infiltration on routine H&E-stained sections. Moreover, considering that FFPE tissue remains one of the main sources of patient material for translational cancer research and the inherent challenges related to the isolation of nucleic acids from FFPE samples^21,22^, we confirmed the ability of CUTseq to yield high-quality SCNA profiles even in old FFPE samples (storage age range: 14–16 years), further highlighting their potential applications. In this cohort of samples, the method successfully detected the loss of 8p, a frequent deletion of breast cancer associated with poor patient survival and cell invasiveness^23^. *MYC*, whose deregulation contributes to BC development and progression and is associated with poor outcomes^24^, represented the most commonly altered gene locus. Not surprisingly, *ERBB2* was also among the most frequently altered loci^25^.

Advances in quantitative IHC and emerging approaches for multiplexed IHC have shifted the landscape of immune profiling from single to multiple marker evaluation^17,26^ in tumor samples. According to a large meta-analysis, multiplexed IHC/IF better predicts benefit to anti-PD-L1/PD-1 treatment over other biomarker modalities (i.e., TMB or gene-expression profiling)^27^, supporting the potential clinical utility of multi-marker assessment. Similarly, machine-learning algorithms and digital image analysis approaches provide the potential for intelligent digital pathology applications, accurate diagnostics and biomarker development^28,29^. The observed correlations between automated assessment of TILs with both manual TIL-WGS scoring^30^ and mfIHC counts, as well as its feasibility in TMAs could pave the way for its widespread use in translational BC studies. Upon validation in larger cohorts, digital image analysis of TILs could greatly facilitate studies on TILs due to the robust reproducibility and low demand in time and manual labor of this technique^31–33^. These high-dimensional methods could also provide the tools for further dissection and deconvolution of tumor microenvironment heterogeneity^34^ and the complex regulation of tumor-host interplay^35,36^. Whether these specific immune-related patterns correlate to clinical outcomes and confer predictive implications for immunotherapy remain to be explored. Similarly, future applications of CUTseq to larger FFPE sample cohorts could enable the identification of clinically relevant SCNA signatures.

The second focus of our study was to explore the association between tumor SCNAs and the host immune response. Following the first tissue/site-agnostic approval of pembrolizumab for patients with microsatellite instability-high or mismatch repair deficient unresectable or metastatic solid tumors^37^, the identification of genetic determinants of anti-tumor immune response^38,39^ is potentially clinically meaningful. TMB could trigger immune response and predict longer survival in BC, despite the generally lower mutational load/immunogenicity and increased heterogeneity of this cancer type^6,40–44^, but its clinical utility still remains questionable. One hypothesis for such association maintains that a greater level of heterogeneity at the genomic and cellular level would lead to a greater number of neoantigens, a stronger immune response and better outcomes. In contrast, it has also been proposed that a low number of infiltrating immune cells causes diminished immune surveillance and immunoediting and thus leads to increased clonal heterogeneity. Of note, aneuploidy and SCNAs involving either larger or smaller (focal) portions of the genome could also drive diverse cancer hallmarks including cell proliferation and immune escape^11,13^ and thus be associated with dismal prognosis^45,46^. Furthermore, SCNAs (especially deletions) in antigen presentation related genes could negatively impact lymphocytic infiltration, irrespective of the TMB, which has been shown to increase the number of neoantigens^13,47^. In this study, we confirmed that a higher SCNA burden is correlated with decreased immune cell infiltration and especially a reduction in stromal TILs. Previous studies have also reported a negative correlation between the SCNA burden and immune gene signatures in BC, mostly in the triple-negative subtype and for whole chromosome/arm aneuploidy as compared to focal SCNAs, further indicating the role of gene dosage effects^13–15,48^. In contrast, a weak positive correlation was noted in two other studies, especially in the ER + subtype^38,49^. Thus, considering (i) the positive correlation of the TMB with SCNAs^13^, the TMB-independent prognostic significance of SCNAs^45^ as well as (iii) the opposing impact on immune infiltration and response to immunotherapy (SCNAs predicted poorer survival in melanoma patients receiving ICB as compared to TMB^13^) the following question arises: Could a combined immunogenomic score including the SCNA burden, TMB and host immune response better stratify BC patients and predict benefit from immunotherapy?

The present exploratory study suffers from several limitations, which will need to be addressed in follow-up studies. Our small sample size did not enable additional subgroup analyses (e.g., further evaluation of predictive implications regarding dose tailored chemotherapy). The few documented events after a 10-year follow-up precluded the exploration of the prognostic value of our findings. Furthermore, the use of TMAs as compared to whole-tissue sections might underestimate the extent of lymphocytic infiltration and the expression of PD-L1/PD-1 axis^50,51^, as indirectly supported by the results of TILs enumeration in our study. Finally, since our study was mostly correlative, causality in our observations remains to be proven.

The present proof-of-principle study indicates the feasibility of SCNA and immune cell profiling applications on a large scale using archival, low-input FFPE BC samples. This could pave the way for further applications and validation in larger patient cohorts, such as the continuation phase III PANTHER trial^52–55^. Our findings also provide insights into the interplay between SCNAs and the immune microenvironment in patients with early BC. Ultimately, we need to identify whether specific immunogenomic patterns confer poor prognosis despite the use of adjuvant therapy, hence marking a candidate population for treatment escalation using immunomodulating therapies.

## Methods

### Study samples

The Scandinavian Breast Group (SBG) 2004-1 study is a randomized phase II study, which aimed to evaluate the feasibility and tolerability of three different adjuvant chemotherapy regimens: (a) tailored according to the hematologic nadirs and dose dense epirubicin, cyclophosphamide and docetaxel (EC → T) every 2 weeks, (b) fixed dose regimen of the same agents every 2 weeks and (c) the TAC regimen (docetaxel, doxorubicin and cyclophosphamide every 3 weeks), enrolling a total of 124 patients with node-positive disease. The patient characteristics and primary safety and efficacy analysis, as well as the long-term follow-up (median: 10.3 years) analysis have been previously reported^56,57^. The analyses performed in the present study have been approved by the ethics committee at Karolinska Institutet, Stockholm, Sweden (Dnr 2017/345-32 and Dnr 2018/1084-32) and by the Swedish Medical Product Agency (Dnr 5.1 2017–51466). Written informed consent was obtained from all patients prior to enrollment in the clinical trial. The conduct of the study conformed to the standards set by the Declaration of Helsinki. This trial was initiated in 2004, when trial registration was not compulsory. It is the feasibility study of a randomized phase III trial (PANTHER, EudraCT number 2007-002061-12 and Clinicaltrials.gov accession number NCT00798070).

### Patient tissue sample processing and tissue microarray construction

FFPE patient tissue was used for this study (storage age range: 14–16 years); an initial whole-tissue section (WTS) (thickness: 4 μm) was taken from each tissue block and subsequently stained with hematoxylin and eosin (H&E) using standard protocols. The tumor area and cellularity (% of tumor cells) were then annotated and confirmed by a certified pathologist (J.H.). Two additional tissue sections (thickness: 10 μm each) from each patient FFPE sample were also obtained only from the tumor area using scalpels directly on the tissue block. These sections were used for DNA extraction as described hereunder. TMA were also constructed from primary tumors of all patients from the FFPE blocks using an automated tissue microarrayer (VTA-100, Veridiam, Oceanside, CA, USA). Each TMA consisted of duplicate cores per tumor/patient (diameter: 1 mm), originating from the previously annotated tumor-rich areas.

### Evaluation of tumor-infiltrating lymphocytes

#### TILs enumeration

Stromal tumor-infiltrating lymphocytes (TIL) were evaluated on H&E-stained full-face sections, by a certified pathologist (J.H.), who was blinded to other clinicopathological and genomic characteristics, as the percentage (%) of tumor stroma covered by infiltrating lymphocytes, according to the recommendations of the International TILs/Immuno-Oncology Biomarker Working Group^30^.

#### Digital-assisted evaluation of TILs

An image-based, automated evaluation of TILs was performed in both H&E-stained whole-slide images (WSI) and TMA, using the QuPath open source software^20,58,59^. Briefly, a classifier algorithm compatible with the QuPath software has been created in order to define tumor cells, lymphocytes, stromal cells and other cells on the stained sections. For both WSI and TMA the following variable easTILs% = TILs Cell Area /Stroma Area*100, was calculated as a surrogate of the respective definition from the TILs Working Group for the visual assessment while different calculated variables/scores for the machine-defined TILs are summarized in the Supplementary Table 4.

### Multiplex fluorescent immunohistochemical staining

Tissue sections (thickness: 4 μm) were prepared from the FFPE blocks of TMA for the staining with multiplex fluorescent immunohistochemistry (mfIHC), enabling the simultaneous and spatial in situ detection of multiple protein markers^60–62^. Specifically, the custom-based 7-color IHC kit (Opal^TM^ 7 Solid Tumor Immunology Kit, Akoya Bioscienes, Malborough, MA, USA), has been optimized in order to include a panel of 6 immune markers: CD4 (1:100, Cat No. M731029-2), CD8a (1:200, Cat No. MA513473), PD-L1 (1:400, Cat No. ab228462), PD-1 (1:100, Cat No. ab52587), FoxP3 (1:300, Cat No. 12653), CD68 (1:400, Cat No. M087629-2). For improved visualization of epithelial tissue, a cocktail of primary antibodies against E-cadherin (1:2000, Cat No. 610182), cytokeratin (1:400, Cat No. GA05361-2) and pan-cytokeratin (1:500, Cat No. ab7753) was used. The detailed protocol and reagent references of the mfIHC procedure in this study are presented in Supplementary Table 5. The fully automated Leica Bond RX^m^ (Leica Biosystems, Buffalo Grove, IL, USA) was used for the multiplex staining. Tissue sections were stained with 4′,6-diamidino-2-phenylindole (DAPI) in order to visualize the nuclei and subsequently mounted with Prolong Diamond Antifade Mountant (ThermoFisher, Waltham, MA, USA).

### Image acquisition, analysis, and thresholding of the multispectral approach

Imaging of the TMA was performed using the Vectra® Polaris™ Automated Quantitative Pathology Imaging System (Akoya Biosciences, Marlborough, MA, USA)^60,61^. A whole-slide scanning (10x) was obtained in order to locate and label the TMA cores using the Phenochart software (Akoya Biosciences, Malborough, MA, USA). Multispectral fluorescent imaging was then applied on the selected regions providing a resolution of 2 pixels per 1 um. Spectral umixing was performed using the inForm^®^ image analysis software (Akoya Biosciences, Malborough, MA, USA) and the signal intensity for each fluorophore was normalized for exposure. A machine-learning tissue segmentation algorithm was then set up and applied for the image analysis following a training step. This included the manual annotation of three distinct compartment/region types (tumor, stroma and blank areas) in a training set of TMA cores. Cell segmentation was carried out based on the nuclear DAPI staining and with the surrounding area (3 mm perinuclear) to be defined as the cytoplasm zone. The established image analysis protocol was applied for the complete set of scanned TMA cores (*n* = 157). Each image was manually evaluated by three investigators (I.Z., A.M., P.M.) so as to exclude necrotic areas, non-tumor tissue and/or staining artifacts from subsequent analyses and discrepancies were resolved by a certified pathologist (A.M.). Both nuclear and cytoplasmic areas (making a total cell area) were assessed for markers’ expression, except FoxP3, which was evaluated only in nuclear areas. The intensity positivity threshold was defined individually for each biomarker using the built-in function for cell phenotyping (inForm^®^ software) in a randomly selected set of TMA cores (20 images, 100 cells visually determined per marker). These thresholds were applied to the output data from the complete cohort to classify cells according to the expression of each marker either as positive or negative. Duplicate TMA cores belonging to the same tumor were merged and cell infiltration was normalized against the total viable tissue area. The cell density (cells/mm^2^) of each single marker, i.e., number of immune marker-positive cells normalized separately to the tumor and stromal areas/compartments was then computed for each sample. Co-expression patterns of the various immune-positive markers were used for the identification of immune cell subpopulations. The analytical workflow of the applied multispectral method is summarized in Fig. 1b.

### DNA extraction from FFPE tumor tissue and blood samples

DNA was extracted from archival FFPE breast cancer patient tissue based on the aforementioned tumor area annotations and description, using the AllPrep DNA/RNA FFPE Kit (Cat. No. 80234, QIAGEN, Germany). Briefly, the main steps of the extraction process involved deparaffinization using xylene, washing in 100% ethanol, air-drying, lysis/digestion using proteinase K, on-column RNAase treatment, genomic DNA binding on QIAmp MinElute spin column, washing and elution of DNA in EB buffer (Cat. No. 19086, QIAGEN, Germany). Germline DNA was extracted also from patients’ peripheral blood samples using the FlexiGene DNA kit (Cat No. 51206, QIAGEN, Germany). Quality control (QC) was performed for the estimated the yield of the extracted DNA. Concentration and the *A*_260_/*A*_280_ and *A*_260/230_ absorbance ratios (purity estimation) were obtained using the spectrophotometer NanoDrop ND-1000 (Saveen Werner, Sweden). Further estimation of the DNA concentration was performed using the Qubit^®^ 3.0 Fluorometer (ThermoFisher Scientific, USA) and the Qubit^™^ dsDNA BR (Broad Range) Assay kit (Cat No Q32850, Invitrogen, USA). Furthermore, the integrity of DNA was estimated based on the DNA Integrity Number (DIN) values, using the Agilent Tapestation 2200 System (Agilent, Santa Clara, CA, USA) according to the manufacturer’s instructions.

### CUTseq

We designed and prepared 96 CUTseq adapters as previously described^16^. 93/96 gDNA FFPE samples and 33/36 gDNA blood samples were used (remaining samples were excluded due to low concentration) and two technical replicates were prepared by two different operators. Upon receival samples were diluted to a concentration of approximately 18 ng/ul. The experiment was performed on a 384-well plate divided into four different parts: (1) top left 96 wells were used for 92 gDNA FFPE samples (replicate 1) and 4 negative control (Nuclease Free Water); (2) top right 96 wells were used for 33 gDNA blood samples (replicate 1) and 3 negative control (Nuclease Free Water); (3) bottom left 96 wells were used for 92 gDNA FFPE samples (replicate 2) and 4 negative control (Nuclease Free Water); (4) bottom right 96 wells were used for 33 gDNA blood samples (replicate 2) and 3 negative control (Nuclease Free Water). First 5 µl of Vapor Lock (Qiagen, Cat.No. 981611) was dispensed in each well to prevent evaporation. After this we used the I-DOT One robot for all subsequent dispensing steps. We dispensed 350 nl of gDNA (concentration around 6.3 ng) from FFPE and blood samples and 350 nl of Nuclease Free Water as control. Digestion mix containing 50 nl of CutSmart buffer (NEB, Cat.no. B7204S) and 100 nl of NlaIII-HF enzyme (NEB, Cat. no. R0125L). Plate was centrifuged at 1200 × *g* for 5 min. Digestion was performed at 37 °C for 30 min followed by 20 min at 65 °C to inactivate the enzyme. After digestion, we dispensed 300 nl of 33 nM CUT adapter together with 700 nl of ligation mix containing 200 nl of T4 rapid DNA ligase (Thermo Fisher Scientific, Cat.No. K1423), 300 nl of T4 ligase buffer (Thermo Fisher Scientific, Cat. No. K1423), 120 nl of 10 mΜ ATP (Thermo Fisher Scientific, Cat.No. PV3227), 30 nl of 50 mg/ml bovine serum albumin (Thermo Fisher Scientific, Cat.No. AM2616), and 50 nl of nuclease-free water (Thermo Fisher Scientific, Cat.No. 4387936) and the plate was incubated at 25 °C for 30 min. After ligation, we added 5 μl of 1xPBS (Thermo Fisher Scientific, Cat.No. AM9625) to each well and pooled the contents of every well (keeping the four different parts separate) with different barcodes in four different eppendorf tubes. From this step the two replicates were performed by two different operators. After short spin, Vapor-Lock (top layer) was removed and purification was carried out by adding 3.7 μl of 20 mg/ml glycogen (Sigma, Cat.No. 10901393001), 11.5 μl of 3 M sodium acetate (Life Technologies, Cat.No. AM9740) and 288 μl of ice-cold Absolute Ethanol (VWR, Cat.No. 20816.367) per 100 μl of DNA solution and incubate at −80 °C overnight. The next day Ethanol precipitation was performed and DNA sonicated using Covaris ME220 Focused-ultrasonicator with the target peak of 200 bp. Sheared DNA was purified with 1.3X ratio of Agencourt Ampure XP beads (Beckman Coulter, Cat. No. A63881). Eight microliters of purified DNA was used as input for the IVT step and library preparation as described in the original CUTseq paper^16^. Final libraries were sequenced on a Illumina NextSeq 500 platform with high output 75 bp single-end kit (Illumina, catalog number FC-404-2005).

### Sequencing data processing and copy number calling

We filtered reads based on the presence of the correct prefix, allowing for 1 mismatch in the restriction site. Following this, we removed the barcode and unique molecular identifier (UMI) from the read and appended to the read header using *umi_tools extract* (version 1.1.1)^63^. Subsequently, we demultiplexed the reads based on the extracted barcodes using *BBMap demuxbyname* (version 38.76) (https://sourceforge.net/projects/bbmap). Demultiplexed reads were then aligned to the human reference genome GRCh37/hg19 using Burrows-Wheeler Aligner (BWA) (version 0.7.17-r1188)^64^. Aligned reads were position sorted and deduplicated with *samtools sort* (version 1.10)^65^ and *umi_tools dedup* (version 1.1.1)^63^, respectively. The preprocessing pipeline used in this study is made available through a snakemake file on https://github.com/ljwharbers/sbg2004-cutseq.

We called somatic copy number variants using a pipeline from the Genome Analysis ToolKit (GATK) (version 4.1.8.0)^66^. In short, we produced a list of annotated intervals using *PreprocessIntervals* and *AnnotateIntervals*. Reads were counted in these intervals using *CollectReadCounts* and a Panel Of Normal of 33 blood samples was created using *CreateReadCountPanelOfNormals*. Tumor profiles were normalized and segments were modeled using *DenoiseReadCounts* and *ModelSegments*, respectively. Finally, significantly amplified or lost segments were called using *CallCopyRatioSegments*. The copy number variant pipeline with the exact parameters used is made available through a snakemake file on https://github.com/ljwharbers/sbg2004-cutseq.

### Data processing and statistical analyses

For comparison of immune cell densities groups of paired samples between tumor and stroma area, the Wilcoxon signed-rank test was used. Descriptive statistics (mean, standard deviation, median) were used for continuous variables (e.g., mfIHC cell densities, eTILs). Unsupervised hierarchical clustering was performed on normalized data in order to identify CNA (based on the called segments of the bins) and/or immune cell clusters. Associations of different immune cell patterns (including TILs expression levels) and DNA copy number alterations with clinicopathological parameters were performed using chi-square (*χ*^2^), Fisher’s exact or Mann–Whitney *U*-tests, where appropriate. The association between TIL enumeration using manual and automated counting was assessed using Spearman correlation. The observed number of survival events in each CNA group was compared using the log-rank test All analyses were performed using SPSS (v.25.0 Corp. Armnok, NY, USA), R studio (version 4.3) and GraphPad Prism (version 8.0. GraphPad software Inc., San Diego, CA, USA).

### Reporting summary

Further information on research design is available in the Nature Research Reporting Summary linked to this article.

## Supplementary information

Reporting Summary

Supplementary Information

Supplementary Data 1

## Data Availability

The segmented SCNA profiles are available in the Supplementary Material (Supplementary Data 1). The raw data files (BAM) and other data that support the findings of this study are available from the corresponding authors (J.B., N.C., T.F.) upon reasonable request and provided that the intended use is in accordance with the ethics approval and the informed consent signed by the trial participants.

## Code availability

All the custom code used to process and analyze the sequencing data is also available at the following GitHub link: https://github.com/ljwharbers/sbg2004-cutseq.
