# Supplementary material for: Interplay between copy number alterations and immune profiles in the early breast cancer Scandinavian Breast Group 2004-1 randomized phase II trial: results from a feasibility study
Source: NPJ Breast Cancer. 2021 Nov 19;7:144. doi: 10.1038/s41523-021-00352-3 (PMC8604966; doi:10.1038/s41523-021-00352-3)
Supplement: Supplementary file 2 — Supplementary Information [file 41523_2021_352_MOESM2_ESM.pdf]

Supplementary Material

Supplementary Figure Legends

Supplementary Figure 1. Mean cell densities of immune cell subpopulations (A) and their co-expression with immune checkpoints (B) in ER-positive and ER-negative tumors

Supplementary Figure 2. Heatmaps of clusters of immune cell subpopulations derived from multiplex IHC using unsupervised hierarchical clustering in stroma (A) and tumor (B) compartments

Supplementary Figure 3. CUTseq experimental replicates' concordance

Supplementary Figure 4. Percentage (A) and size (B) of the altered genome (amplified or deleted) in ER-positive and ER-negative tumors. In the boxplots, each box extends from the 25th to the 75th percentile, the midline represents the median, and the whiskers extend from –1.5×IQR to +1.5×IQR from the closest quartile, where IQR is the inter-quartile range

Supplementary Tables

Supplementary Table 1. Cell densities (cells/mm^2) of the different immune cell subsets as derived from the multiplex fluorescent IHC method

Supplementary Table 2. Correlation matrix values for the different immune cell subsets

Supplementary Table 3. Patient characteristics per manual TILs group in the SBG-2004-1 study cohort

Supplementary Table 4. Variables derived from the digital TILs evaluation

Supplementary Table 5. List of antibodies used for the multiplex fluorescent IHC method

Supplementary Data 1. Segmented Somatic Copy Number Alterations profiles by CUTseq

Supplementary Figure 1

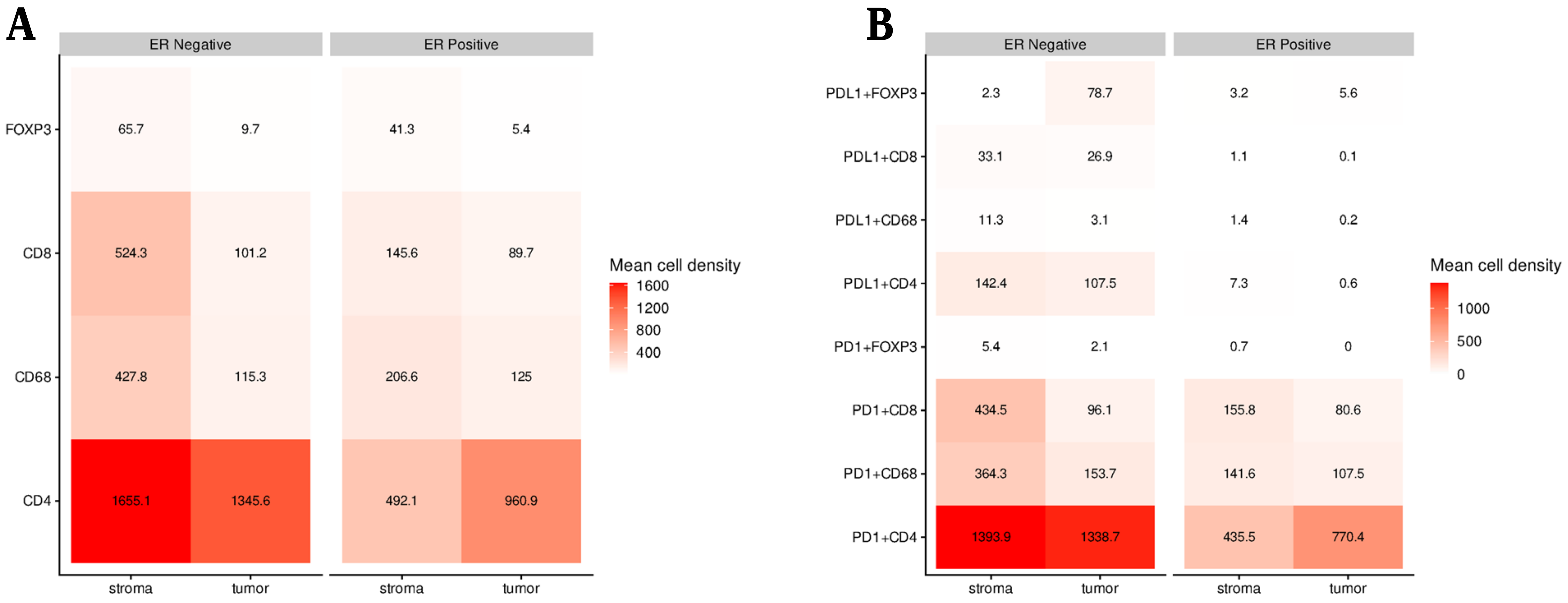

Supplementary Figure 2

A) Stroma compartment

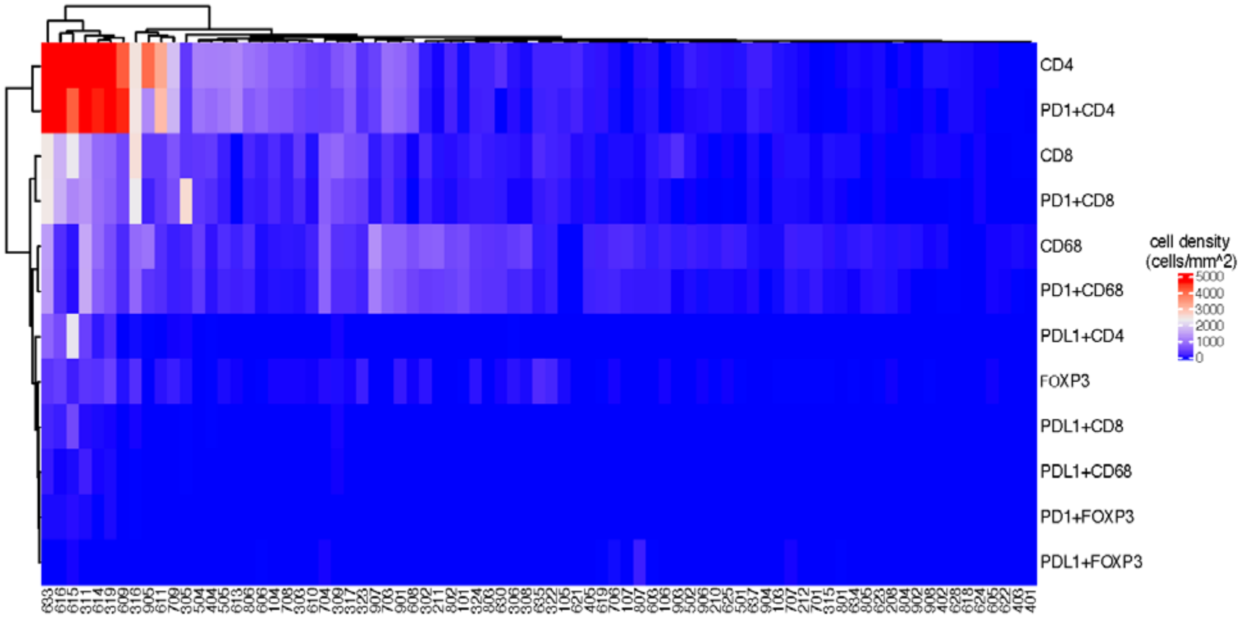

B) Tumor compartment

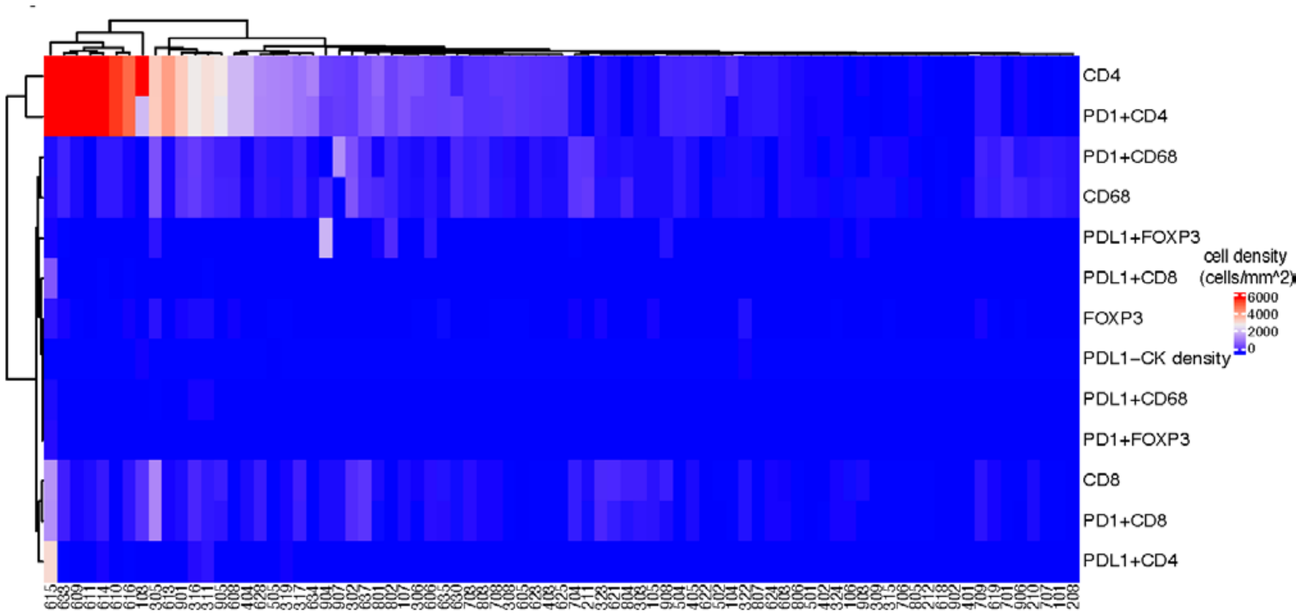

Supplementary Figure 3

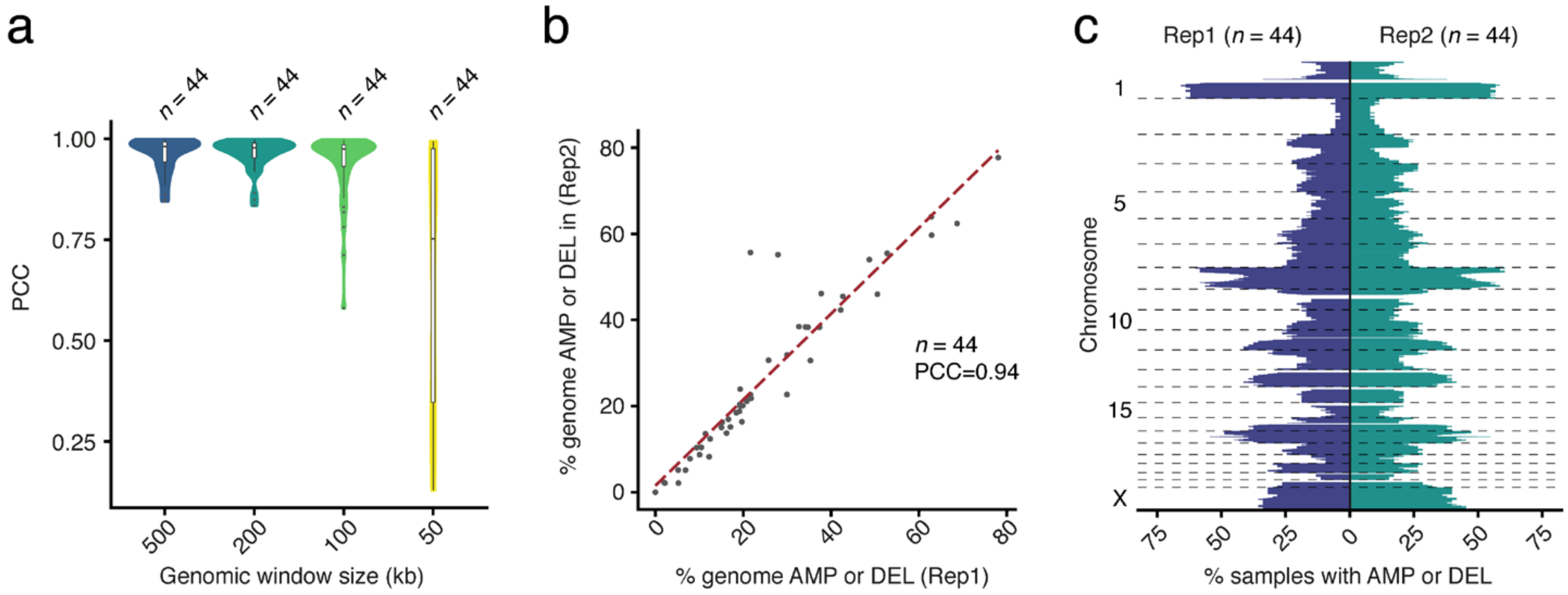

Supplementary Figure 4

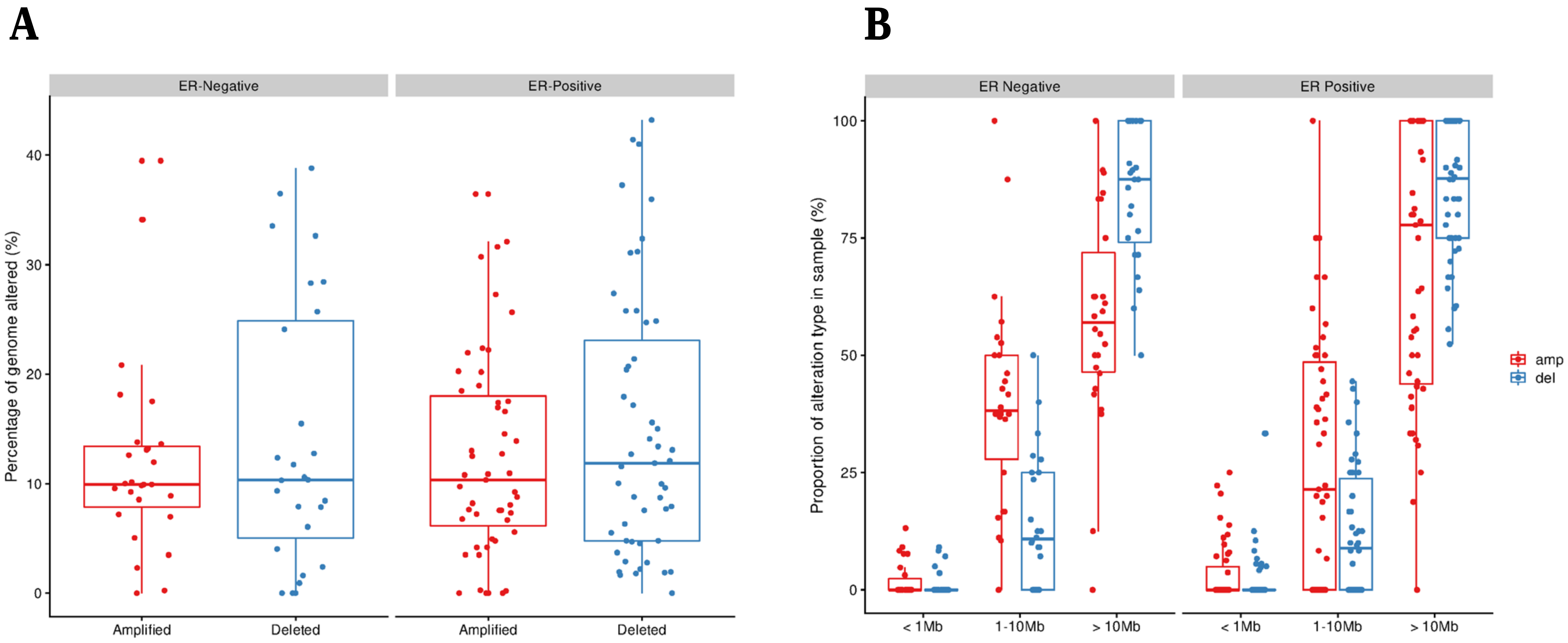

Supplementary Table 1. Cell densities (cells/mm^2) of the different immune cell subsets as derived from the multiplex fluorescent IHC method (n=79)

**Cell type**	**Median**	**Mean**	**SD**	**p-value[§]**
CD4+ cells				
intra-tumoral	271	1104	1967	0.4541
stromal	141.8	909	1860	
CD8+ cells				
intra-tumoral	23.87	91.46	228.2	<0.0001 ****
stromal	81.3	278.8	510.9	
CD68+ cells				
intra-tumoral	59.32	114.2	136.1	<0.0001 ****
stromal	146.9	278.3	329.1	
FOXP3+ cells				
intra-tumoral	0	6.9	16.84	<0.0001 ****
stromal	0	49.56	95.97	
PD-1+CD4+ cells				
intra-tumoral	285.8	976.4	1606	0.0432 *
stromal	86.87	779.6	1649	
PD-1+CD8+ cells				
intra-tumoral	22.28	83.8	222.7	<0.0001 ****
stromal	40.27	254.8	522.2	
PD-1+CD68+ cells				
intra-tumoral	52.44	117	197.1	<0.0001 ****
stromal	112.6	217.5	302.1	
PD-1+FOXP3+ cells				
intra-tumoral	0	0.75	6.2	0.0195 *
stromal	0	2.3	8.8	
PD-L1+CD4+ cells				
intra-tumoral	0	38.52	320.5	0.0080 **
stromal	0	55.25	269.1	
PD-L1+CD8+ cells				
intra-tumoral	0	9.58	83.94	0.0840
stromal	0	12.45	63.07	
PD-L1+CD68+ cells				
intra-tumoral	0	1.25	5.63	0.0923
stromal	0	4.87	21.37	
PD-L1+FOXP3+ cells				
intra-tumoral	0	31.55	212.3	0.2221
stromal	0	2.88	16.22	

[§]Wilcoxon sign-rank test

Supplementary Table 2. Correlation matrix values for the different immune cell subsets

	eTILs (WSI)	etTILs (WSI)	esTILs (WSI)	easTILs (WSI)	eTILs (TMA)	etTILs (TMA)	esTILs (TMA)	easTILs (TMA)	TIL	CD4 (Stroma)	CD68 (Stroma)	CD8 (Stroma)	FOXP3 (Stroma)	FOXP3+CD4+CD8 (Stroma)	FOXP3+CD4 (Stroma)	FOXP3+CD68 (Stroma)	FOXP3+CD8 (Stroma)	PD1+CD4 (Stroma)	PD1+CD68 (Stroma)	PD1+CD8 (Stroma)	PD1+FOXP3 (Stroma)	PDL1+CD4 (Stroma)	PDL1+CD68 (Stroma)	PDL1+CD8 (Stroma)	PDL1+FOXP3 (Stroma)	CD4 (Tumor)	CD68 (Tumor)	CD8 (Tumor)	FOXP3 (Tumor)	FOXP3+CD4+CD8 (Tumor)	FOXP3+CD4 (Tumor)	FOXP3+CD68 (Tumor)	FOXP3+CD8 (Tumor)	PD1+CD4 (Tumor)	PD1+CD68 (Tumor)	PD1+CD8 (Tumor)	PD1+FOXP3 (Tumor)	PDL1+CD4 (Tumor)	PDL1+CD68 (Tumor)	PDL1+CD8 (Tumor)	PDL1+FOXP3 (Tumor)
eTILs (WSI)	1	[illegible]	[illegible]	[illegible]	[illegible]	[illegible]	[illegible]	[illegible]	[illegible]	0.457479508196721	[illegible]	[illegible]	[illegible]	[illegible]	[illegible]	[illegible]	[illegible]	[illegible]	[illegible]	[illegible]	[illegible]	[illegible]	[illegible]	[illegible]	[illegible]	[illegible]	[illegible]	[illegible]	[illegible]	[illegible]	[illegible]	[illegible]	[illegible]	[illegible]	[illegible]	[illegible]	[illegible]	[illegible]	[illegible]	[illegible]	[illegible]
etTILs (WSI)	0.916068989071038	1	[illegible]	[illegible]	[illegible]	[illegible]	[illegible]	[illegible]	[illegible]	0.522284836065574	[illegible]	[illegible]	[illegible]	[illegible]	[illegible]	[illegible]	[illegible]	[illegible]	[illegible]	[illegible]	[illegible]	[illegible]	[illegible]	[illegible]	[illegible]	[illegible]	[illegible]	[illegible]	[illegible]	[illegible]	[illegible]	[illegible]	[illegible]	[illegible]	[illegible]	[illegible]	[illegible]	[illegible]	[illegible]	[illegible]	[illegible]
esTILs (WSI)	[illegible]	[illegible]	1	0.871243169398907	[illegible]	[illegible]	[illegible]	[illegible]	[illegible]	0.414190573770492	[illegible]	[illegible]	[illegible]	[illegible]	[illegible]	[illegible]	[illegible]	[illegible]	[illegible]	[illegible]	[illegible]	[illegible]	[illegible]	[illegible]	[illegible]	[illegible]	[illegible]	[illegible]	[illegible]	[illegible]	[illegible]	[illegible]	[illegible]	[illegible]	[illegible]	[illegible]	[illegible]	[illegible]	[illegible]	[illegible]	[illegible]
easTILs (WSI)	[illegible]	[illegible]	[illegible]	1	[illegible]	[illegible]	[illegible]	[illegible]	[illegible]	0.543630464480874	[illegible]	[illegible]	[illegible]	[illegible]	[illegible]	[illegible]	[illegible]	[illegible]	[illegible]	[illegible]	[illegible]	[illegible]	[illegible]	[illegible]	[illegible]	[illegible]	[illegible]	[illegible]	[illegible]	[illegible]	[illegible]	[illegible]	[illegible]	[illegible]	[illegible]	[illegible]	[illegible]	[illegible]	[illegible]	[illegible]	[illegible]
eTILs (TMA)	[illegible]	[illegible]	[illegible]	[illegible]	1	[illegible]	[illegible]	[illegible]	[illegible]	0.364951974718881	[illegible]	[illegible]	[illegible]	[illegible]	[illegible]	[illegible]	[illegible]	[illegible]	[illegible]	[illegible]	[illegible]	[illegible]	[illegible]	[illegible]	[illegible]	[illegible]	[illegible]	[illegible]	[illegible]	[illegible]	[illegible]	[illegible]	[illegible]	[illegible]	[illegible]	[illegible]	[illegible]	[illegible]	[illegible]	[illegible]	[illegible]
etTILs (TMA)	[illegible]	[illegible]	[illegible]	[illegible]	0.946981985827713	1	[illegible]	[illegible]	[illegible]	0.36559231623494	[illegible]	[illegible]	[illegible]	[illegible]	[illegible]	[illegible]	[illegible]	[illegible]	[illegible]	[illegible]	[illegible]	[illegible]	[illegible]	[illegible]	[illegible]	[illegible]	[illegible]	[illegible]	[illegible]	[illegible]	[illegible]	[illegible]	[illegible]	[illegible]	[illegible]	[illegible]	[illegible]	[illegible]	[illegible]	[illegible]	[illegible]
esTILs (TMA)	[illegible]	[illegible]	[illegible]	[illegible]	[illegible]	[illegible]	1	[illegible]	[illegible]	0.283842049351485	[illegible]	[illegible]	[illegible]	[illegible]	[illegible]	[illegible]	[illegible]	[illegible]	[illegible]	[illegible]	[illegible]	[illegible]	[illegible]	[illegible]	[illegible]	[illegible]	[illegible]	[illegible]	[illegible]	[illegible]	[illegible]	[illegible]	[illegible]	[illegible]	[illegible]	[illegible]	[illegible]	[illegible]	[illegible]	[illegible]	[illegible]
easTILs (TMA)	[illegible]	[illegible]	[illegible]	[illegible]	[illegible]	[illegible]	[illegible]	1	[illegible]	0.313340448524575	[illegible]	[illegible]	[illegible]	[illegible]	[illegible]	[illegible]	[illegible]	[illegible]	[illegible]	[illegible]	[illegible]	[illegible]	[illegible]	[illegible]	[illegible]	[illegible]	[illegible]	[illegible]	[illegible]	[illegible]	[illegible]	[illegible]	[illegible]	[illegible]	[illegible]	[illegible]	[illegible]	[illegible]	[illegible]	[illegible]	[illegible]
TIL	[illegible]	[illegible]	[illegible]	[illegible]	[illegible]	[illegible]	[illegible]	[illegible]	1	0.451561837144576	[illegible]	[illegible]	[illegible]	[illegible]	[illegible]	[illegible]	[illegible]	[illegible]	[illegible]	[illegible]	[illegible]	[illegible]	[illegible]	[illegible]	[illegible]	[illegible]	[illegible]	[illegible]	[illegible]	[illegible]	[illegible]	[illegible]	[illegible]	[illegible]	[illegible]	[illegible]	[illegible]	[illegible]	[illegible]	[illegible]	[illegible]
CD4 (Stroma)	[illegible]	[illegible]	[illegible]	[illegible]	[illegible]	[illegible]	[illegible]	[illegible]	[illegible]	1	[illegible]	[illegible]	[illegible]	[illegible]	[illegible]	[illegible]	[illegible]	[illegible]	[illegible]	[illegible]	[illegible]	[illegible]	[illegible]	[illegible]	[illegible]	[illegible]	[illegible]	[illegible]	[illegible]	[illegible]	[illegible]	[illegible]	[illegible]	[illegible]	[illegible]	[illegible]	[illegible]	[illegible]	[illegible]	[illegible]	[illegible]
CD68 (Stroma)	[illegible]	[illegible]	[illegible]	[illegible]	[illegible]	[illegible]	[illegible]	[illegible]	[illegible]	0.391991120218579	1	[illegible]	[illegible]	[illegible]	[illegible]	[illegible]	[illegible]	[illegible]	[illegible]	[illegible]	[illegible]	[illegible]	[illegible]	[illegible]	[illegible]	[illegible]	[illegible]	[illegible]	[illegible]	[illegible]	[illegible]	[illegible]	[illegible]	[illegible]	[illegible]	[illegible]	[illegible]	[illegible]	[illegible]	[illegible]	[illegible]
CD8 (Stroma)	[illegible]	[illegible]	[illegible]	[illegible]	[illegible]	[illegible]	[illegible]	[illegible]	[illegible]	0.788422131147541	0.497267759562842	1	[illegible]	[illegible]	[illegible]	[illegible]	[illegible]	[illegible]	[illegible]	[illegible]	[illegible]	[illegible]	[illegible]	[illegible]	[illegible]	[illegible]	[illegible]	[illegible]	[illegible]	[illegible]	[illegible]	[illegible]	[illegible]	[illegible]	[illegible]	[illegible]	[illegible]	[illegible]	[illegible]	[illegible]	[illegible]
FOXP3 (Stroma)	[illegible]	[illegible]	[illegible]	[illegible]	[illegible]	[illegible]	[illegible]	[illegible]	[illegible]	0.452019394862411	[illegible]	[illegible]	1	[illegible]	[illegible]	[illegible]	[illegible]	[illegible]	[illegible]	[illegible]	[illegible]	[illegible]	[illegible]	[illegible]	[illegible]	[illegible]	[illegible]	[illegible]	[illegible]	[illegible]	[illegible]	[illegible]	[illegible]	[illegible]	[illegible]	[illegible]	[illegible]	[illegible]	[illegible]	[illegible]	[illegible]
FOXP3+CD4+CD8 (Stroma)	[illegible]	[illegible]	[illegible]	[illegible]	[illegible]	[illegible]	[illegible]	[illegible]	[illegible]	0.497055566455658	[illegible]	[illegible]	[illegible]	1	[illegible]	[illegible]	[illegible]	[illegible]	[illegible]	[illegible]	[illegible]	[illegible]	[illegible]	[illegible]	[illegible]	[illegible]	[illegible]	[illegible]	[illegible]	[illegible]	[illegible]	[illegible]	[illegible]	[illegible]	[illegible]	[illegible]	[illegible]	[illegible]	[illegible]	[illegible]	[illegible]
FOXP3+CD4 (Stroma)	[illegible]	[illegible]	[illegible]	[illegible]	[illegible]	[illegible]	[illegible]	[illegible]	[illegible]	0.666941798240382	[illegible]	[illegible]	[illegible]	[illegible]	1	[illegible]	[illegible]	[illegible]	[illegible]	[illegible]	[illegible]	[illegible]	[illegible]	[illegible]	[illegible]	[illegible]	[illegible]	[illegible]	[illegible]	[illegible]	[illegible]	[illegible]	[illegible]	[illegible]	[illegible]	[illegible]	[illegible]	[illegible]	[illegible]	[illegible]	[illegible]
FOXP3+CD68 (Stroma)	[illegible]	[illegible]	[illegible]	[illegible]	[illegible]	[illegible]	[illegible]	[illegible]	[illegible]	0.321155185947289	[illegible]	[illegible]	[illegible]	[illegible]	[illegible]	1	[illegible]	[illegible]	[illegible]	[illegible]	[illegible]	[illegible]	[illegible]	[illegible]	[illegible]	[illegible]	[illegible]	[illegible]	[illegible]	[illegible]	[illegible]	[illegible]	[illegible]	[illegible]	[illegible]	[illegible]	[illegible]	[illegible]	[illegible]	[illegible]	[illegible]
FOXP3+CD8 (Stroma)	[illegible]	[illegible]	[illegible]	[illegible]	[illegible]	[illegible]	[illegible]	[illegible]	[illegible]	0.420275141427198	[illegible]	[illegible]	[illegible]	[illegible]	[illegible]	[illegible]	1	[illegible]	[illegible]	[illegible]	[illegible]	[illegible]	[illegible]	[illegible]	[illegible]	[illegible]	[illegible]	[illegible]	[illegible]	[illegible]	[illegible]	[illegible]	[illegible]	[illegible]	[illegible]	[illegible]	[illegible]	[illegible]	[illegible]	[illegible]	[illegible]
PD1+CD4 (Stroma)	[illegible]	[illegible]	[illegible]	[illegible]	[illegible]	[illegible]	[illegible]	[illegible]	[illegible]	0.988387978142077	[illegible]	[illegible]	[illegible]	[illegible]	[illegible]	[illegible]	[illegible]	1	[illegible]	[illegible]	[illegible]	[illegible]	[illegible]	[illegible]	[illegible]	[illegible]	[illegible]	[illegible]	[illegible]	[illegible]	[illegible]	[illegible]	[illegible]	[illegible]	[illegible]	[illegible]	[illegible]	[illegible]	[illegible]	[illegible]	[illegible]
PD1+CD68 (Stroma)	[illegible]	[illegible]	[illegible]	[illegible]	[illegible]	[illegible]	[illegible]	[illegible]	[illegible]	0.473531420765027	[illegible]	[illegible]	[illegible]	[illegible]	[illegible]	[illegible]	[illegible]	[illegible]	1	[illegible]	[illegible]	[illegible]	[illegible]	[illegible]	[illegible]	[illegible]	[illegible]	[illegible]	[illegible]	[illegible]	[illegible]	[illegible]	[illegible]	[illegible]	[illegible]	[illegible]	[illegible]	[illegible]	[illegible]	[illegible]	[illegible]
PD1+CD8 (Stroma)	[illegible]	[illegible]	[illegible]	[illegible]	[illegible]	[illegible]	[illegible]	[illegible]	[illegible]	0.810553504144365	[illegible]	[illegible]	[illegible]	[illegible]	[illegible]	[illegible]	[illegible]	[illegible]	[illegible]	1	[illegible]	[illegible]	[illegible]	[illegible]	[illegible]	[illegible]	[illegible]	[illegible]	[illegible]	[illegible]	[illegible]	[illegible]	[illegible]	[illegible]	[illegible]	[illegible]	[illegible]	[illegible]	[illegible]	[illegible]	[illegible]
PD1+FOXP3 (Stroma)	[illegible]	[illegible]	[illegible]	[illegible]	[illegible]	[illegible]	[illegible]	[illegible]	[illegible]	0.515779241059786	[illegible]	[illegible]	[illegible]	[illegible]	[illegible]	[illegible]	[illegible]	[illegible]	[illegible]	[illegible]	1	[illegible]	[illegible]	[illegible]	[illegible]	[illegible]	[illegible]	[illegible]	[illegible]	[illegible]	[illegible]	[illegible]	[illegible]	[illegible]	[illegible]	[illegible]	[illegible]	[illegible]	[illegible]	[illegible]	[illegible]
PDL1+CD4 (Stroma)	[illegible]	[illegible]	[illegible]	[illegible]	[illegible]	[illegible]	[illegible]	[illegible]	[illegible]	0.708635299163849	[illegible]	[illegible]	[illegible]	[illegible]	[illegible]	[illegible]	[illegible]	[illegible]	[illegible]	[illegible]	[illegible]	1	[illegible]	[illegible]	[illegible]	[illegible]	[illegible]	[illegible]	[illegible]	[illegible]	[illegible]	[illegible]	[illegible]	[illegible]	[illegible]	[illegible]	[illegible]	[illegible]	[illegible]	[illegible]	[illegible]
PDL1+CD68 (Stroma)	[illegible]	[illegible]	[illegible]	[illegible]	[illegible]	[illegible]	[illegible]	[illegible]	[illegible]	0.453998291698206	[illegible]	[illegible]	[illegible]	[illegible]	[illegible]	[illegible]	[illegible]	[illegible]	[illegible]	[illegible]	[illegible]	[illegible]	1	[illegible]	[illegible]	[illegible]	[illegible]	[illegible]	[illegible]	[illegible]	[illegible]	[illegible]	[illegible]	[illegible]	[illegible]	[illegible]	[illegible]	[illegible]	[illegible]	[illegible]	[illegible]
PDL1+CD8 (Stroma)	[illegible]	[illegible]	[illegible]	[illegible]	[illegible]	[illegible]	[illegible]	[illegible]	[illegible]	0.549774709096945	[illegible]	[illegible]	[illegible]	[illegible]	[illegible]	[illegible]	[illegible]	[illegible]	[illegible]	[illegible]	[illegible]	[illegible]	[illegible]	1	[illegible]	[illegible]	[illegible]	[illegible]	[illegible]	[illegible]	[illegible]	[illegible]	[illegible]	[illegible]	[illegible]	[illegible]	[illegible]	[illegible]	[illegible]	[illegible]	[illegible]
PDL1+FOXP3 (Stroma)	[illegible]	[illegible]	[illegible]	[illegible]	[illegible]	[illegible]	[illegible]	[illegible]	[illegible]	0.111462182890902	[illegible]	[illegible]	[illegible]	[illegible]	[illegible]	[illegible]	[illegible]	[illegible]	[illegible]	[illegible]	[illegible]	[illegible]	[illegible]	[illegible]	1	[illegible]	[illegible]	[illegible]	[illegible]	[illegible]	[illegible]	[illegible]	[illegible]	[illegible]	[illegible]	[illegible]	[illegible]	[illegible]	[illegible]	[illegible]	[illegible]
CD4 (Tumor)	[illegible]	[illegible]	[illegible]	[illegible]	[illegible]	[illegible]	[illegible]	[illegible]	[illegible]	0.553545925251761	[illegible]	[illegible]	[illegible]	[illegible]	[illegible]	[illegible]	[illegible]	[illegible]	[illegible]	[illegible]	[illegible]	[illegible]	[illegible]	[illegible]	[illegible]	1	[illegible]	[illegible]	[illegible]	[illegible]	[illegible]	[illegible]	[illegible]	[illegible]	[illegible]	[illegible]	[illegible]	[illegible]	[illegible]	[illegible]	[illegible]
CD68 (Tumor)	[illegible]	[illegible]	[illegible]	[illegible]	[illegible]	[illegible]	[illegible]	[illegible]	[illegible]	0.176400273224044	[illegible]	[illegible]	[illegible]	[illegible]	[illegible]	[illegible]	[illegible]	[illegible]	[illegible]	[illegible]	[illegible]	[illegible]	[illegible]	[illegible]	[illegible]	[illegible]	1	[illegible]	[illegible]	[illegible]	[illegible]	[illegible]	[illegible]	[illegible]	[illegible]	[illegible]	[illegible]	[illegible]	[illegible]	[illegible]	[illegible]
CD8 (Tumor)	[illegible]	[illegible]	[illegible]	[illegible]	[illegible]	[illegible]	[illegible]	[illegible]	[illegible]	0.394081737492761	[illegible]	[illegible]	[illegible]	[illegible]	[illegible]	[illegible]	[illegible]	[illegible]	[illegible]	[illegible]	[illegible]	[illegible]	[illegible]	[illegible]	[illegible]	[illegible]	[illegible]	1	[illegible]	[illegible]	[illegible]	[illegible]	[illegible]	[illegible]	[illegible]	[illegible]	[illegible]	[illegible]	[illegible]	[illegible]	[illegible]
FOXP3 (Tumor)	[illegible]	[illegible]	[illegible]	[illegible]	[illegible]	[illegible]	[illegible]	[illegible]	[illegible]	0.370533795806122	[illegible]	[illegible]	[illegible]	[illegible]	[illegible]	[illegible]	[illegible]	[illegible]	[illegible]	[illegible]	[illegible]	[illegible]	[illegible]	[illegible]	[illegible]	[illegible]	[illegible]	[illegible]	1	[illegible]	[illegible]	[illegible]	[illegible]	[illegible]	[illegible]	[illegible]	[illegible]	[illegible]	[illegible]	[illegible]	[illegible]
FOXP3+CD4+CD8 (Tumor)	[illegible]	[illegible]	[illegible]	[illegible]	[illegible]	[illegible]	[illegible]	[illegible]	[illegible]	0.283094727874555	[illegible]	[illegible]	[illegible]	[illegible]	[illegible]	[illegible]	[illegible]	[illegible]	[illegible]	[illegible]	[illegible]	[illegible]	[illegible]	[illegible]	[illegible]	[illegible]	[illegible]	[illegible]	[illegible]	1	[illegible]	[illegible]	[illegible]	[illegible]	[illegible]	[illegible]	[illegible]	[illegible]	[illegible]	[illegible]	[illegible]
FOXP3+CD4 (Tumor)	[illegible]	[illegible]	[illegible]	[illegible]	[illegible]	[illegible]	[illegible]	[illegible]	[illegible]	0.467383734907851	[illegible]	[illegible]	[illegible]	[illegible]	[illegible]	[illegible]	[illegible]	[illegible]	[illegible]	[illegible]	[illegible]	[illegible]	[illegible]	[illegible]	[illegible]	[illegible]	[illegible]	[illegible]	[illegible]	[illegible]	1	[illegible]	[illegible]	[illegible]	[illegible]	[illegible]	[illegible]	[illegible]	[illegible]	[illegible]	[illegible]
FOXP3+CD68 (Tumor)	[illegible]	[illegible]	[illegible]	[illegible]	[illegible]	[illegible]	[illegible]	[illegible]	[illegible]	0.30104602433321	[illegible]	[illegible]	[illegible]	[illegible]	[illegible]	[illegible]	[illegible]	[illegible]	[illegible]	[illegible]	[illegible]	[illegible]	[illegible]	[illegible]	[illegible]	[illegible]	[illegible]	[illegible]	[illegible]	[illegible]	[illegible]	1	[illegible]	[illegible]	[illegible]	[illegible]	[illegible]	[illegible]	[illegible]	[illegible]	[illegible]
FOXP3+CD8 (Tumor)	[illegible]	[illegible]	[illegible]	[illegible]	[illegible]	[illegible]	[illegible]	[illegible]	[illegible]	0.358543017637736	[illegible]	[illegible]	[illegible]	[illegible]	[illegible]	[illegible]	[illegible]	[illegible]	[illegible]	[illegible]	[illegible]	[illegible]	[illegible]	[illegible]	[illegible]	[illegible]	[illegible]	[illegible]	[illegible]	[illegible]	[illegible]	[illegible]	1	[illegible]	[illegible]	[illegible]	[illegible]	[illegible]	[illegible]	[illegible]	[illegible]
PD1+CD4 (Tumor)	[illegible]	[illegible]	[illegible]	[illegible]	[illegible]	[illegible]	[illegible]	[illegible]	[illegible]	0.549445901424574	[illegible]	[illegible]	[illegible]	[illegible]	[illegible]	[illegible]	[illegible]	[illegible]	[illegible]	[illegible]	[illegible]	[illegible]	[illegible]	[illegible]	[illegible]	[illegible]	[illegible]	[illegible]	[illegible]	[illegible]	[illegible]	[illegible]	[illegible]	1	[illegible]	[illegible]	[illegible]	[illegible]	[illegible]	[illegible]	[illegible]
PD1+CD68 (Tumor)	[illegible]	[illegible]	[illegible]	[illegible]	[illegible]	[illegible]	[illegible]	[illegible]	[illegible]	0.38849043715847	[illegible]	[illegible]	[illegible]	[illegible]	[illegible]	[illegible]	[illegible]	[illegible]	[illegible]	[illegible]	[illegible]	[illegible]	[illegible]	[illegible]	[illegible]	[illegible]	[illegible]	[illegible]	[illegible]	[illegible]	[illegible]	[illegible]	[illegible]	[illegible]	1	[illegible]	[illegible]	[illegible]	[illegible]	[illegible]	[illegible]
PD1+CD8 (Tumor)	[illegible]	[illegible]	[illegible]	[illegible]	[illegible]	[illegible]	[illegible]	[illegible]	[illegible]	0.418164747563832	[illegible]	[illegible]	[illegible]	[illegible]	[illegible]	[illegible]	[illegible]	[illegible]	[illegible]	[illegible]	[illegible]	[illegible]	[illegible]	[illegible]	[illegible]	[illegible]	[illegible]	[illegible]	[illegible]	[illegible]	[illegible]	[illegible]	[illegible]	[illegible]	[illegible]	1	[illegible]	[illegible]	[illegible]	[illegible]	[illegible]
PD1+FOXP3 (Tumor)	[illegible]	[illegible]	[illegible]	[illegible]	[illegible]	[illegible]	[illegible]	[illegible]	[illegible]	0.377318794017877	[illegible]	[illegible]	[illegible]	[illegible]	[illegible]	[illegible]	[illegible]	[illegible]	[illegible]	[illegible]	[illegible]	[illegible]	[illegible]	[illegible]	[illegible]	[illegible]	[illegible]	[illegible]	[illegible]	[illegible]	[illegible]	[illegible]	[illegible]	[illegible]	[illegible]	[illegible]	1	[illegible]	[illegible]	[illegible]	[illegible]
PDL1+CD4 (Tumor)	[illegible]	[illegible]	[illegible]	[illegible]	[illegible]	[illegible]	[illegible]	[illegible]	[illegible]	0.516983120512468	[illegible]	[illegible]	[illegible]	[illegible]	[illegible]	[illegible]	[illegible]	[illegible]	[illegible]	[illegible]	[illegible]	[illegible]	[illegible]	[illegible]	[illegible]	[illegible]	[illegible]	[illegible]	[illegible]	[illegible]	[illegible]	[illegible]	[illegible]	[illegible]	[illegible]	[illegible]	[illegible]	1	[illegible]	[illegible]	[illegible]
PDL1+CD68 (Tumor)	[illegible]	[illegible]	[illegible]	[illegible]	[illegible]	[illegible]	[illegible]	[illegible]	[illegible]	0.505888917095066	[illegible]	[illegible]	[illegible]	[illegible]	[illegible]	[illegible]	[illegible]	[illegible]	[illegible]	[illegible]	[illegible]	[illegible]	[illegible]	[illegible]	[illegible]	[illegible]	[illegible]	[illegible]	[illegible]	[illegible]	[illegible]	[illegible]	[illegible]	[illegible]	[illegible]	[illegible]	[illegible]	[illegible]	1	[illegible]	[illegible]
PDL1+CD8 (Tumor)	[illegible]	[illegible]	[illegible]	[illegible]	[illegible]	[illegible]	[illegible]	[illegible]	[illegible]	0.44797090763344	[illegible]	[illegible]	[illegible]	[illegible]	[illegible]	[illegible]	[illegible]	[illegible]	[illegible]	[illegible]	[illegible]	[illegible]	[illegible]	[illegible]	[illegible]	[illegible]	[illegible]	[illegible]	[illegible]	[illegible]	[illegible]	[illegible]	[illegible]	[illegible]	[illegible]	[illegible]	[illegible]	[illegible]	[illegible]	1	[illegible]
PDL1+FOXP3 (Tumor)	[illegible]	[illegible]	[illegible]	[illegible]	[illegible]	[illegible]	[illegible]	[illegible]	[illegible]	-0.0530624534363702	[illegible]	[illegible]	[illegible]	[illegible]	[illegible]	[illegible]	[illegible]	[illegible]	[illegible]	[illegible]	[illegible]	[illegible]	[illegible]	[illegible]	[illegible]	[illegible]	[illegible]	[illegible]	[illegible]	[illegible]	[illegible]	[illegible]	[illegible]	[illegible]	[illegible]	[illegible]	[illegible]	[illegible]	[illegible]	[illegible]	1

Supplementary Table 3. Patient characteristics per manual TILs group (median cut-off)

	Study cohort	High TILs	Low TILs	P value
	**N = 89 (%)**	**N = 43 (%)**	**N = 46 (%)**	
**Age**<br>Median (min – max)	51.1 (28.7 – 64.7)	50.8 (31.0 – 64.2)	51.8 (28.7 – 64.7)	0.895*
**Tumor Size mm**<br>Median (min – max)	24 (2 – 150)	21 (2 – 121)	27.50 (9 – 150)	0.264*
**Positive lymph nodes**<br>Median (min – max)	6 (1 – 35)	5 (1 – 35)	7.5 (1 – 16)	0.581*
**Grade**				
1	11 (12.4)	2 (4.7)	9 (19.6)	
2	31 (34.8)	9 (20.9)	22 (47.8)	**<0.001****
3	47 (52.8)	32 (74.4)	15 (32.6)	
**Estrogen Receptor**				
Positive	57 (64.0)	18 (41.9)	39 (84.8)	**<0.001****
Negative	32 (36.0)	25 (58.1)	7 (15.2)	
**Progesterone Receptor**				
Positive	40 (44.9)	15 (34.9)	25 (54.3)	0.065**
Negative	49 (55.1)	28 (65.1)	21 (45.7)	
**HER2**				
Positive	27 (30.3)	14 (32.6)	13 (28.3)	
Negative	60 (67.4)	28 (65.1)	32 (69.6)	0.203**
Unknown	2 (2.2)	1 (2.3)	1 (2.2)	

* Mann Whitney U test

** Pearson's x^2

Supplementary Table 4. Definition of variables derived from digital tumor- infiltrating lymphocytes (TILs) evaluation

Variable	Definition
**eTILs%**	100* TILs/Sum of Tumor Cells and TILs
**etTILs%** *(total)*	100* TILs/ Sum of TILs, Tumor cells, fibroblast and others
**esTILs%** *(stromal)*	100* TILs/Sum of TILs, fibroblast and others adjacent to tumor
**eaTILs** *(cell density)*	Sum of TILs/Sum of areas of tumor region analyzed **(mm^2)**
**easTILs%**	TILs Cell Area /Stroma Area*100
**Manual/visual stromal TILs** *(sTIL)*	The percentage (%) of tumour stroma covered by infiltrating lymphocytes (International TILs Working Group)

Supplementary Table 5. List of antibodies used for the multiplex fluorescent IHC method

Order	Antigen retrieval*	Marker	Clone	Host Species	Dilution	Company
1.	pH9	CD68	PG-M1	Mouse	1:400	Agilent
2.	pH6	PD-1	NAT105	Mouse	1:100	Abcam
3.	pH6	CD4	4B12	Mouse	1:100	Agilent
4.	pH6	CD8a	C8/144B	Mouse	1:200	Thermo Fisher
5.	pH6	PD-L1	SP142	Rabbit	1:400	Abcam
6.	pH6	FoxP3	D6O8R	Rabbit	1:300	Cell Signaling
7.	pH6	PanCK	C-11	Mouse	1:500	Abcam
		Cytokeratin	AE1/AE3	Mouse	1:400	Dako
		E-cahderin	36/E	Mouse	1:2000	BD Biosciences
8.	-	DAPI	-	-	-	Perkin Elmer

*****Antigen retrieval performed in an automated Leica Bond RX^{m} Research Stainer at 95 ^{0}C, 20min.
The ImmPRESS® HRP Anti-Mouse IgG (Peroxidase) (Cat. No: MP-7402-50) and Anti-Rabbit IgG (Peroxidase) Polymer Detection Kits, made in Horse (Cat No: MP-7401-50) (Vector Laboratories) were used as secondary antibodies
